# Disposition and effects of alpha-pyrrolidinoisohexanophenone (α-PHiP) in comparison with cocaine: an observational study

**DOI:** 10.3389/fphar.2025.1728824

**Published:** 2026-01-14

**Authors:** Georgina De la Rosa, Giorgia Iacobone, Esther Papaseit, Olga Hladun, Lourdes Poyatos, Dolly Andrea Caicedo, Martha Catalina Argote, Soraya Martín, Mireia Ventura, Alessandro Di Giorgi, Silvia Graziano, Simona Pichini, Emilia Marchei, Magí Farré, Clara Pérez-Mañá

**Affiliations:** 1 Clinical Pharmacology Department, Hospital Universitari Germans Trias i Pujol and Institut de Recerca Germans Trias i Pujol (HUGTP-IGTP), Badalona, Spain; 2 Department of Pharmacology, Therapeutics and Toxicology, Universitat Autònoma de Barcelona, Barcelona, Spain; 3 National Center on Addiction and Doping, National Institute of Health, Rome, Italy; 4 Department of Chemistry, Faculty of Mathematical, Physical and Natural Science, ‘Sapienza’ University of Rome, Rome, Italy; 5 Department of Medicine and Life Sciences, Universitat Pompeu Fabra, Barcelona, Spain; 6 Drug Addiction Unit. Parc de Salut Mar, Hospital del Mar Research Institute, Barcelona, Spain; 7 Energy Control, Associació Benestar i Desenvolupament, Barcelona, Spain; 8 Laboratorio Antidoping FMSI, Federazione Medico Sportiva Italiana, Rome, Italy

**Keywords:** cocaine, pharmacokinetics, psychostimulants, synthetic cathinones, alpha-pyrrolidinoisohexanophenone (α‐PHiP)

## Abstract

**Introduction:**

Alpha-pyrrolidinoisohexanophenone (α-PHiP) is a synthetic cathinone structurally related to alpha-pyrrolidinopentiophenone (α-PVP) and other pyrrolidinophenones, acting as a potent monoamine transporter inhibitor. Despite its raising prevalence on the recreational drug market, limited data exist about its human pharmacology. This study aimed to assess its concentrations in different matrices and characterize its acute effects in humans in comparison with cocaine when administered by intranasal route.

**Methods:**

A non-controlled observational study was conducted under naturalistic conditions in seventeen healthy adults (nine men, eight women) with prior experience using psychostimulants. Participants self-administered intranasal doses of 15–25 mg α-PHiP or 75–90 mg cocaine in two separate sessions. Physiological parameters and subjective effects (VAS, ARCI, and VESSPA-SSE questionnaires) were assessed repeatedly at baseline and up to 5 h post-administration. Biological samples (oral fluid, urine, sweat), were collected to evaluate concentration-time profiles, including a time point of dried blood spot.

**Results:**

α-PHiP was rapidly absorbed, reaching maximum concentrations in oral fluid at 20 min and showed a short half-life (1.1 h). The amount excreted unchanged in urine or present in sweat was less than 1%. Dried blood spot levels showed high interindividual variability in the range of intoxication cases. Both α-PHiP and cocaine increased heart rate while the increase in blood pressure was more sustained for α-PHiP. Subjective effects of α-PHiP peaked within the first hour and were very similar to those of cocaine, being the feelings of high and intensity reported lower.

**Conclusion:**

This study provides novel insights into the effects and distribution of α-PHiP across various biological matrices. α-PHiP elicited prototypical subjective effects characteristic of psychostimulants, confirming its potential for abuse. Compared to cocaine, α-PHiP produced similar subjective effects. Future research should further investigate the detection of its metabolites to improve monitoring and understanding of its pharmacokinetics.

## 1 Introduction

The setting of new psychoactive substances (NPS) continues to evolve rapidly, presenting significant challenges for public health and forensic toxicology. Among these, synthetic cathinones represent a wide and diverse class of compounds known for their stimulant and empathogenic effects (WHO, 2022; Glennon and Dukat, 2017). Synthetic cathinones were developed from cathinone, an alkaloid present in the leaves of the shrub *Catha edulis* (Khat, qat, or cat) (Valente et al., 2014), to enhance the stimulant properties and modify the pharmacological effects of the parent compound. The aim was to produce more potent or long-lasting effects while circumventing existing legal restrictions on natural cathinone derivatives (Banks et al., 2014).

Alpha-pyrrolidinoisohexanophenone (α-PHiP or α-PiHP) is a synthetic cathinone belonging to the pyrrolidinophenone subclass. This subclass is also known as pyrovalerone derivatives and includes compounds such as 3,4-methylenedioxypyrovalerone (MDPV) and α-pyrrolidinopentiophenone (α-PVP). These substances share a core cathinone structure and are characterized by the incorporation of a pyrrolidine ring into the side chain (Dinis et al., 2024). This modification increases lipophilicity and enhances their potency at monoamine transporters, particularly those for dopamine (DAT) and norepinephrine (NET) (Dinis et al., 2024; Baarset et al., 2025).

The mechanism of action of α-PHiP involves potent inhibition of dopamine and norepinephrine reuptake, with a potency comparable to that of α-PVP at the dopamine transporter (DAT). However, some authors have reported slightly greater potency for α-PVP (Persson et al., 2024). In contrast, α-PHiP shows slightly lower potency at the norepinephrine transporter (NET). Neither compound exhibits significant inhibition of 5-HT reuptake. This mechanism is analogous to that of cocaine, where the psychostimulant effect results from the inhibition of synaptic reuptake rather than increased neurotransmitter release (Baarset et al., 2025).

These findings suggest a high potential for abuse, given that DAT inhibition is strongly linked to pronounced psychostimulant and reinforcing effects (Glennon and Dukat, 2017; Persson et al., 2024; Nadal-Gratacós et al., 2024; Kolaczynska et al., 2021). α-PHiP is a positional isomer of α-pyrrolidinohexiophenone (α-PHP), which was first identified by the European Monitoring Centre for Drugs and Drug Addiction in 2016 (EMCDDA, 2016). Since then, α-PHiP has gained increasing relevance in the new psychoactive substances (NPS) market, as reflected by its growing prevalence in seizures and forensic case reports (Dinis et al., 2024). This escalating presence underscores the urgent need for comprehensive understanding of its pharmacological effects, pharmacokinetics and disposition in biological matrices (WHO, 2022).

While there is published information on the analysis, pharmacology, and metabolites of some synthetic cathinones as a group, as well as on the clinical symptoms of intoxication and treatment in humans (Coppola and Mondola, 2012; Fujita et al., 2018; Pieprzyca et al., 2022; Chen et al., 2024), information on α-PHiP remains very limited (Adamowicz et al., 2020; Wachholz et al., 2023; Dinis et al., 2024). In user surveys, α-PHiP has been reported to be consumed by oral, intravenous, rectal, and intranasal routes, with typical intranasal doses ranging from 10 to 25 mg (Dinis et al., 2024). No previous naturalistic observational studies or clinical trials in humans have been published with this drug (search strategy in Pubmed: “alpha-pyrrolidinoisohexanophenone OR α-PHiP OR α-PiHP OR pyrrolidine cathinone. Limits: Human”).

Comprehensive drug disposition across multiple biological matrices provide valuable insights into the pharmacokinetics (absorption, distribution, metabolism, and excretion) of substance. Each biological matrix offers distinct advantages for drug monitoring, particularly in the context of NPS detection. Moreover, the detection window varies depending on the biological sample used and can be influenced by several factors, such as the frequency and dosage of drug intake, as well as individual physiological characteristics. Generally, for short detection windows blood, including dried blood samples (DBS), or oral fluid (OF) are the usual matrices, urine and sweat are more suitable for medium detection windows, while hair and nails are the preferred choices for long-term detection (Dinis-Oliveira et al., 2016; Øiestad et al., 2016; de Campos et al., 2022).

This study aimed to investigate the pharmacological effects and disposition of α-PHiP in multiple human biological matrices (OF, urine, sweat and DBS) and using cocaine as a control drug, following the intranasal self-administration by healthy consumers. Cocaine was selected because both drugs are central nervous system stimulants that act as inhibitors on monoamine transporters, particularly dopamine transporter (although α-PHiP is reported to be a more potent DAT inhibitor than cocaine, WHO, 2022). Due to that, their pharmacological profiles and behavioral effects are expected to be similar. Furthermore, both substances are commonly used intranasally in recreational settings and may be also co-consumed to enhance additive or synergistic effects.

By addressing the existing knowledge gaps regarding α-PHiP pharmacological and toxicological profile, our research will support forensic interpretation, enhance analytical detection methods, and contribute to clinical toxicology and public health initiatives concerning NPS.

## 2 Materials and methods

### 2.1 Participants

Seventeen healthy adults (nine men and eight women) voluntarily participated in the study. Inclusion criteria required participants to be of either gender, aged between 18 and 45 years, without significant medical conditions or ongoing treatments that could interfere with the study drug’s safety or pharmacokinetics, with no history of psychiatric disorders, and with previous experience with recreational intranasal use of psychostimulants and/or synthetic cathinones. Subjects older than 45 years were excluded because this group of substances are used between 18 and 40 years and to avoid the possible progressive natural physiological and pharmacokinetic changes related to aging and to increase safety. Exclusion criteria included a history of significant medical or psychiatric disorders (with the exception of nicotine use disorder), prior severe adverse reactions to psychostimulants, current long-term medication use, and pregnancy.

Participants were recruited through word of mouth in collaboration with Energy Control (Asociación Bienestar y Desarrollo, https://energycontrol.org/), a harm-reduction non-government organization (NGO) that provides counseling and drug-checking services. The study protocol was approved by the local Human Research Ethics Committee (CEIC HUGTiP, Badalona, Spain; ref. PI-18–267). All participants received detailed information about the study’s objectives and procedures and provided written informed consent prior to any study-related activity. The study was conducted in accordance with the Declaration of Helsinki and applicable Spanish research regulations (Biomedical Research Law 14/2007). Participants were financially compensated for their time and involvement.

### 2.2 Design conditions

We conducted a prospective, non-controlled observational study in a naturalistic setting. The study design, including procedures and assessment methods, was consistent with those applied in our previous research on the acute effects of other NPS (De la Rosa et al., 2025; Poyatos et al., 2021; Papaseit et al., 2021; Martínez et al., 2021; Papaseit et al., 2020a). Participants independently acquired the substance from unidentified suppliers, and the products were analysed by Energy Control. Participants were allowed to select their own doses within predefined ranges based on common use patterns reported in the literature: 75–90 mg for cocaine and 15–25 mg for α-PHiP. The selected doses were determined according to participants’ prior consumption experience. Specifically, five male participants selected 90 mg of cocaine, while three female participants chose 75 mg. For α-PHiP, five females and one male selected 15 mg, whereas three males opted for 25 mg.

### 2.3 Procedures

Participants were asked to abstain from consuming any recreational substances during the week prior to the selection visit, and to avoid alcohol, energy drinks and caffeinated beverages within 24 h before the experimental session, in order to minimize the risk of interactions or residual effects. After a selection visit, which was used to confirm eligibility, two experimental sessions (one for each substance) were conducted. During the selection visit, participants were asked to provide information on their medical history (including mental health disorders), drug consumption history, and underwent a physical examination and a urine drug test. The selection visit took place 2–3 days before the session.

On the day of the session, participants were asked to arrive at 15:00 at a private club in Barcelona, with the aim to simulate a recreational consumption environment. The study setting was closed to the public. Each session had a duration of 5 h, beginning immediately after the self-administration of the selected substance.

Upon arrival, participants were required to provide a urine for a drug screening test to confirm abstinence from prior substance use. The screening tested for the presence of benzodiazepines, barbiturates, morphine, methadone, cocaine, amphetamines, methamphetamine, MDMA, THC, and tricyclic antidepressants using the Drug-Screen Multi 10TD Test (Multi-Line, Nal Von Minden, Moers, Germany). Previous alcohol intake was ruled out by assessing breath alcohol concentration (Drager Alcotest 5820, Dragerwerk AG & Co., Lübeck, Germany). In addition, Female participants underwent a urine pregnancy test (Glip Test Plus hCG Card®, Ref 30,701, Biosynex, Delémont, Switzerland), which was required to be negative for inclusion in the study.

During the session, participants were allowed to engage in leisure activities of their choice, such as listening to music, dancing, or conversing. Assessments were performed at baseline (prior to self-administration) and along 5 h after intranasal self-administration of α-PHiP or cocaine. A light snack (a piece of fruit) was provided 2 h after administration.

At each time point, assessments followed a predefined sequence: i) oral fluid (OF) sample collection (from 5 min to the scheduled time point), ii) measurement of vital signs (from 5 min to the scheduled time point) and iii) completion of questionnaires (from the scheduled time point until 5 min later). Capillary blood at 1 h and urine samples at 2 h and 5 h following administration were collected after the completion of the questionnaires.

In both sessions OF samples were collected using Salivette tubes (Sarstedt AG & Co. KG, Nümbrecht, Germany) before and following substance self-consumption at 0.33 h (20 min) and 0.66 h (40 min), 1, 1.5, 2, 2.5, 3, 4, and 5 h. Samples were centrifuged after collection and stored at −20 °C until analysis. Urine was collected in two different time ranges: 0–2 and 2–5 h following consumption. The total volume was registered, and a sample was collected in 1.5-mL tubes and stored at −20 °C prior to analysis. Sweat collection patches (Cosmopor E, Hartmann, Heidenheim, Germany) were applied to the backs of the subjects, under the scapula, 4 h before administration (−4 to 0 h) and up to 5 h after self-administration (0–5 h). Dry blood samples were collected via finger prick (Whatman filter paper, Maidstone, UK), where a blood drop filling a pre-marked circle corresponded to approximately 30 µL of blood**.** DBS samples were collected 1 h after consumption and stored at ambient temperature until analysis**.**

### 2.4 Physiological and subjective effects

Noninvasive heart rate (HR), systolic blood pressure (SBP), diastolic blood pressure (DBP) and cutaneous temperature were measured using an automatic monitor (Omron, Hoofdorp, Netherlands) with subjects seated at baseline, and at 20 min, 40 min, 1 h, 1.5 h, 2 h, 2.5 h, 3 h, 4 h, and 5 h after administration.

Subjective effects were assessed at baseline, 0.33 h (20 min), 0.66 h (40 min), 1 h, 1.5 h, 2 h, 2.5 h, 3 h, 4 h, and 5 h after administration, using a series of visual analog scales (VASs) and the Addiction Research Center Inventory_ARCI 49 item-short form (same time points except 20 min). The Evaluation of Subjective Effects of Substances with Abuse Potential questionnaire (VESSPA-SSE) was administered at baseline, 1, 2, 3, 4, and 5 h. The different tools were administered in the following order (VASs, ARCI and VESSPA). Additionally, the Positive and Negative Syndrome Scale for Schizophrenia (PANSS) was administered at baseline and at 5 h (De la Rosa et al., 2025; Poyatos et al., 2023).

### 2.5 Quantification of α-PHiP in biological matrices

α-PHiP and α-Pyrrolidinovalerophenone-d8 (α-PVP-d8) as internal standard (IS) were purchased from Cayman Chemicals (Ann Arbor, MI, USA). LC-MS grade water and analytical grade ethyl acetate and other chemicals (sodium phosphate and sodium hydrogen phosphate, sodium carbonate and sodium hydrogen carbonate) were obtained from Carlo Erba (Cornaredo, Italy).

Working standard solutions of α-PHiP (10, 1 and 0.1 μg/mL) and the IS (10 and 1 μg/mL) solution were prepared by appropriate methanolic dilution of stock solution. OF calibrators were prepared by adding appropriate volumes of the working standard solutions to blank OF to cover a calibration range of 5–1,000 ng/mL. Blank urine and DBS samples were spiked in the range of 5–500 ng/mL, while blank sweat patches was spiked in the range of 5–500 ng/patch.

Low- and medium-quality control (QC) samples were prepared at concentrations of 7.5 and 250 ng/mL or ng/patch, respectively, OF, urine, DBS and sweat patches samples. High-QC samples were prepared at 850 ng/mL for OF, and at 450 ng/mL or ng/patch, for urine, DBS, and sweat patches samples, respectively.

A similar extraction procedure was carried out for all matrices. Aliquots of (50 µL), urine (100 µL), DBS (30 µL), and sweat (with the absorbent pad removed from the patch using clean tweezers) were transferred into clean glass tubes and spiked with the appropriate volume of IS working solution. Specifically, 5, 10, and 3 µL of a 1 μg/mL IS solution were added to saliva, urine, and DBS samples, respectively, while 10 µL of a 10 μg/mL IS solution were added to sweat samples, to achieve a final concentration of 100 ng/mL or ng/patch for saliva, urine, DBS, and sweat, respectively.

After the addition of 200 µL (for OF and urine) or 1.5 mL (for sweat and DBS) of a carbonate/bicarbonate buffer solution (0.8 M, pH 9.0), sweat and DBS samples were sonicated for 10 min prior to liquid–liquid extraction (LLE) with 1 mL of ethyl acetate using a horizontal shaker for 10 min. OF and urine samples were subjected directly to LLE. Following extraction, the samples were centrifuged at 4,000 rpm for 5 min. The organic phase was collected and evaporated to dryness under a gentle stream of nitrogen. The resulting residues were reconstituted in 50 µL of ethyl acetate, transferred to autosampler vials, and 0.5 µL was injected into the GC-MS/MS system for α-PHiP quantification.

Analysis was performed on a 7890B GC system equipped with a multimode inlet (MMI) and a 7693A Automatic Sampler (all from Agilent Technologies, Santa Clara, CA, USA). The capillary column used was an Agilent Technologies HP-5MS UI (30 m × 0.25 mm × 0.25 m). The samples were injected in pulsed splitless mode with the injector port temperature of 260 °C. Helium was used as a quenching gas at a flow of 2.25 mL/min The column temperature was initially set at 80 °C for 1 min before increasing to 160 °C at 20 °C/min, to 250 °C at 10 °C/min and then increasing again to 290 °C at 35 °C/min (held for 1 min), taking the total run time to 16.14 min.

The GC instrument was interfaced to a 7000D GC/MS triple quadrupole mass spectrometer (Agilent Technologies, Santa Clara, CA, USA). The MS/MS analyses were conducted in positive electron ionization (EI) mode. The transfer line and ion source temperature were set at 280  C. Nitrogen was used as the collision-induced dissociation (CID) gas for ion fragmentation at a flow of 1.5 mL/min.

Quantifications were performed using multiple reaction monitoring (MRM) transitions. Two transitions for each analyte and one transition for the deuterated standards were selected. The α-PHiP quantifier transition was m/z 140 > 98 (Collision energy: 14 eV) and its qualifier transition was 140 > 84 m/z (Collision energy: 10 eV), while α-PVP-d8 transition was 134 > 105 m/z (Collision energy: 14 eV).

Prior to application to real samples, the analytical method was assessed for linearity, sensitivity, accuracy, precision, carryover, and dilution integrity in accordance with the guidelines established by the Organization of Scientific Area Committees for Forensic Science (OSAC, 2019). The validation protocol encompassed the evaluation of bias, precision (within-run and between-run), calibration model, limit of detection (LOD), lower limit of quantification (LLOQ), carryover, matrix interferences, and dilution integrity, as previously described (Di Trana A et al., 2025).

In brief, linearity was assessed using six calibration curves, each comprising six points, over five different days. The acceptance criteria required quantification within ±15% of the target, a coefficient of determination (*R* ^2^) ≥ 0.99, and a quantifier/qualifier ratio within ±20%. Additionally, the Mandel test was performed to confirm linearity. Sensitivity was evaluated by determining the LOD and LLOQ. The LOD was defined as the lowest concentration showing a peak within ±0.1 min of the average retention time and a signal-to-noise ratio ≥3, using diluted fortified blank matrices. The LLOQ was established at the lowest non-zero calibrator, with retention time and quantification within ±0.1 min and ±20%, respectively.

Bias was calculated as the percent deviation of the mean measured concentration from the nominal value across five independent runs at three concentration levels (low, medium, and high QC). An acceptable bias was within ±20%. Precision was expressed as the coefficient of variation (%CV) and evaluated by triplicate analysis of QC samples across five runs within the same day and over 5 days. A %CV <20% was considered acceptable. Carryover was assessed by injecting blank matrix samples immediately following the highest calibrator. The method was deemed free of carryover if no analyte signal with a signal-to-noise ratio ≥3 appeared within ±0.1 min of the calibrator retention time.

Dilution integrity was verified by analysing samples spiked above the upper calibration limit (up to 20-fold) and diluted to fall within the validated range. A % CV <20% was considered acceptable.

The quantitative methods for α-PHiP were successfully validated (see Supplementary Table 1). Each assessed parameter proved to be within the acceptable criteria proposed by OSAC for Forensic Sciences guidelines (OSAC, 2019).

### 2.6 Quantification of cocaine and benzoylecgonine in biological matrices

Aliquots OF (50 µL), urine (50 µL), and sweat (with the absorbent pad removed from the patch using clean tweezers) were transferred into clean glass tubes and spiked with the appropriate volume of IS working solution to achieve a concentration of 100 ng/mL or ng/patch for OF, urine, and sweat, respectively.

Following the addition of 500 µL of phosphate buffer pH 6.0 for OF and urine, or 2 mL for sweat, all samples underwent solid-phase extraction (SPE) using Strata™-X-C cation exchange cartridges (Phenomenex, Torrance, CA, USA). Cartridges were conditioned with 2 mL of methanol, 2 mL of water, and 2 mL of phosphate buffer (pH 6.0). Samples were then loaded, washed sequentially with 2 mL of water and 1 mL of 0.1 M HCl, followed by 2 mL of methanol, and finally eluted with 2 mL of a dichloromethane/isopropanol/ammonium hydroxide mixture (77:20:3, v/v/v). The eluates were evaporated to dryness and derivatized with 50 µL of N, O Bis(trimethylsilyl)trifluoroacetamide solution with 1% of trimethylchlorosilane (BSTFA+ 1%TMS) in a heating block at 70 °C for 30 min prior to GC-MS/MS analysis. 1 μL was injected to Cocaine (COC) and its metabolite benzoylecgonine (BZE) determination**.**

COC and BZE were detected using a previously published and validated gas chromatography–tandem mass spectrometry (GC-MS/MS) method (La Maida et al., 2021). The analytes were separated by gas chromatography and detected using a triple quadrupole mass spectrometer operating in multiple reaction monitoring (MRM) mode with positive electron ionization. MRM transitions were 182→82 (collision energy 10 eV) and 182→108 (collision energy 10 eV) for COC, and 361→82 (collision energy 20 eV) and 240→82 (collision energy 20 eV) for BZE. Underline transition was used for the quantification.

The method was validated (see Supplementary Table 2) according to international guidelines and demonstrated acceptable performance in terms of linearity (*R* ^2^ ≥ 0.990). The limits of quantification (LOQ) were 10 ng/mL or ng/patch and 1 ng/mL or ng/patch for COC and BZE, respectively and adequate for the purpose of the present study. Intra- and inter-assay precision were always below 20.0%, and accuracy deviations did not exceed ±10.3%. Dilution integrity was assessed, and over-curve sample concentrations remained within ±15% of the target values for all analytes.

### 2.7 Quantification of cortisol in oral fluid

In the OF samples, cortisol levels were also quantified to explore potential stress-related or metabolic interactions with α-PHiP and cocaine exposure. Salivary cortisol was quantified using electrochemiluminescence immunoassay (ECLIA) method (Elecsys Cortisol II), performed on the Cobas e analyzer platform (Roche Diagnostics GmbH, Mannheim, Germany).

### 2.8 Statistical analysis

The determination of the sample size was based on the methodology of bioequivalence studies, which resulted in eight to nine subjects per substance needed, considering an alpha risk of 0.05, a power of 80%, and a difference of at least 35% between 20 min and baseline values in the intensity/high effect, considering a 25% of variability.

Peak concentration (C_max_) and time taken to reach peak concentration (T_max_) in OF were directly obtained from the concentration–time curves. The area under the curve (AUC_0–5h_) was calculated by the linear trapezoidal rule. The terminal-phase elimination half-life (t_1/2_) was calculated as 0.693/Ke, where Ke (elimination rate constant) was the slope of the apparent elimination phase of the natural logarithmic (ln) transformation of the OF concentration–time curve, which was estimated using linear regression.

Differences with respect to baseline were calculated for vital signs and subjective effects. Maximum effects (E_max_) and the time needed to reach maximum effects (T_max_) were also calculated for these outcomes. The areas under the curve of the effects (AUC_0–5h_) were calculated also using the trapezoidal rule.

Two-way analysis of variance (ANOVA) was conducted to evaluate the influence of dose and gender/sex on the OF concentrations and effects (E_max_ and AUC values). A gender/sex effect was observed in several PHiP subjective outcomes (32% of them had higher values in men who received a higher dose). Due to our limited sample size, we decided to include all the participants in one group to compare with cocaine, and we provide the gender differences in a supplementary table (see Supplementary Table 3).

The comparison between the effects of α-PHiP and cocaine was performed with an independent sample T-test (E_max_ and AUC). We also conducted a Dunnett *post hoc* test to compare the different time points with baseline values, which was adjusted for multiple comparisons. T_max_ values were compared with the non-parametric Mann-Whitney U-test.

Statistical analysis was carried out using PASW Statistics version 18 (SPSS Inc., Chicago, IL, United States). Differences were considered statistically significant when the resulting p value was <0.05.

## 3 Results

### 3.1 Participants

A total of 17 subjects (nine males and eight females) participated in the study. Their mean age was 31.82 ± 5.96 years (range: 24–46 years), mean weighted 66.04 ± 12.78 kg (range: 44–80 kg), and mean body mass index (BMI) was 22.46 ± 3.47 kg/m^2^ (range: 20–30 kg/m^2^).

All selected participants reported prior experience with psychostimulants, including cathinones, cocaine, MDMA, amphetamines, methamphetamines and 4-bromo-2,5- dimethoxyphenethylamine (2-CB). They reported a median of 218 (range 27–1,156) lifetime uses, a median of 51 (range 3–255) uses in the past year and a median of 4 (range 0–18) uses in the past month. Six were current tobacco smokers, and all reported alcohol consumption. At the beginning of the experimental session, all participants tested negative on urine drug screening, and no clinical signs of intoxication were observed at baseline.

### 3.2 Physiological, subjective effects and adverse effects

**FIGURE 1:**
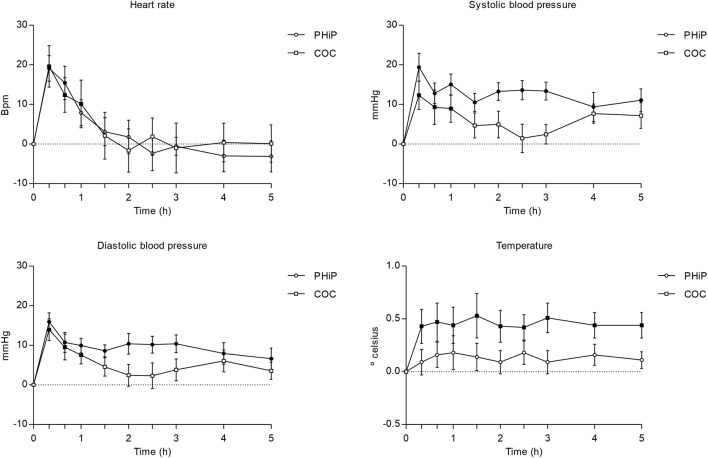
Time course (n = 17; mean ± standard error) of physiological effects (heart rate and blood pressure and temperature) following intranasal administration of α-PHiP (PHiP in the legend) and cocaine. Significant differences from the baseline are indicated with filled symbols • (p < 0.05).

The summary of the parameters of physiological outcomes can be seen in Table 1. Furthermore, it includes statistically significant comparisons to baseline using the Dunnett test for the different time assessments. Additionally, Figure 1 shows the time course of heart rate and systolic blood pressure.

**TABLE 1:** Summary of results on physiological measures.

Physiological effects	Parameters	Mean ± SD		T-student	Dunnet’s test	
​	​	α-PHiP	Cocaine	*p*-value	α-PHiP	Cocaine
SBP (mmHg)	E_max_	23.25 ± 6.29	15.43 ± 7.90	0.053	**a.b.c**.d.**e.f.g**.h.**i**	**a.b**.c
	T_max_	0.33 (0.33–1.00)	0.49 (0.33–1.00)	0.541		
	AUC_0–5h_	60.67 ± 25.13	29.57 ± 36.93	0.058		
DBP (mmHg)	E_max_	17.72 ± 5.98	15.56 ± 6.67	0.493	**a.b.c.d.e.f.g.h**.i	**a.b**.c
	T_max_	0.33 (0.33–2.00)	0.33 (0.33–1.00)	0.236		
	AUC_0–5h_	46.65 ± 27.43	26.33 ± 31.21	0.174		
HR (bpm)	E_max_	21.55 ± 9.41	21.25 ± 14.80	0.960	**a.b**	**a.b**.c
	T_max_	0,33 (0,33–0,66)	0.33 (0.33–0.66)	0.277		
	AUC_0–5 h_	11.09 ± 45.10	15.74 ± 68.85	0.870		
Temperature (°C)	E_max_	0.27 ± 0.48	0.68 ± 0.51	0.116	NS	**a.b.c.d.e.f.g.h.i**
	T_max_	1.00 (0.33–4.00)	1.5 (1.00–2.50)	0.673		
	AUC_0–5 h_	0.63 ± 1.54	2.21 ± 1.90	0.078		
Cortisol (µg/dL)	E_max_	0.12 ± 0.27 (*n* = 8)	0.08 ± 0.02 (*n* = 6)	0.742	NS (*n* = 8)	NS (*n* = 6)
	T_max_	1.75 (0.66–4.00)	2 (1.50–4.00)	0.573		
	AUC_0–5 h_	0.05 ± 0.40	−0.21 ± 0.36	0.238		

E_max_, peak effects 0–5 h (differences from baseline) measured by mmHg (systolic blood pressure [SBP], diastolic blood pressure [DBP]), bpm (heart rate [HR]), °C (temperature [T]). A posthoc Dunnett’s test for multiple comparisons was used. Statistical differences are presented as “a” p < 0.05, “**a**” p < 0.01 (times 0–0.33 h), “b” p < 0.05, “**b**” p < 0.01 (times 0–0.66 h), “c” p < 0.05, “**c**” p < 0.01 (times 0–1 h), “d” p < 0.05, “**d**“ p < 0.01 (times 0–1.5 h), “e” p < 0 .05, “**e**” p < 0.01 (times 0–2 h) and “f” p < 0.05, “**f**” p < 0.01 (times 0–2.5 h), “g” p<0.05 “**g**” p < 0.01 (times 0–3 h), “h” p < 0.05, “**h**” p < 0.01 (times 0–4 h) and “i” p<0.05, “**i**” p<0.01 (times 0–5 h). For T_max_ data are reported, as median and range.

Regarding vital signs, both substances produced a significant increase in heart rate compared to baseline, with a trend for a higher increase with the synthetic cathinone. α-PHiP induced a sustained and statistically significant elevation of SBP over the 5 h, whereas for cocaine it was observed during the first hour. A comparable pattern was observed for diastolic blood pressure, showing also similar peak values among both substances.

Finally, no significant effect on body temperature was detected after α-PHiP administration, while cocaine produced a sustained increase across the 5-h monitoring period.

Table 2 presents the pharmacodynamic parameters for all the subjective outcomes. α-PHiP and cocaine produced psychostimulant subjective effects collected through VAS, ARCI and VESSPA-SSE. Overall, subjects reported subjective effects starting at 20 min with maximum values within the first hour. Subjective effects of both substances disappeared from 3 to 5 h. Although the maximum effects were similar, cocaine produced greater intensity and more pronounced high effects than α-PHiP. The synthetic cathinone mainly produced good effects, liking, clarity, focused, open to others, trust to others and would like to be with other people.

**TABLE 2:** Summary of results on subjective effects.

Subjective effects	Parameters	Mean ± SD		T-student	Dunnet’s test	
​	​	α-PHiP	Cocaine	*p* value	α-PHiP	Cocaine
VAS intensity (mm)	E_max_	22.78 ± 10.59	43.22 ± 23.77	**0.028**	**a.b**.**c**.d	**a.b.c**
	T_max_	0.33 (0.33–1.50)	0.50 (0.33–1.00)	0.673		
	AUC_0–5 h_	25.38 ± 17.34	43.22 ± 23.77	0.095		
VAS stimulated (mm)	E_max_	29.89 ± 14.85	50.88 ± 26.49	0.059	**a.b**.**c**	**a.b.c**
	T_max_	0.33 (0.33–0.66)	0.33 (0.33–0.66)	0.541		
	AUC_0–5 h_	32.04 ± 25.01	46.75 ± 26.77	0.260		
VAS high (mm)	E_max_	25.89 ± 10.45	50.25 ± 28.47	**0.030**	**a.b**.**c**	**a.b.c**
	T_max_	0.33 (0.33–1.00)	0.33 (0.33–0.66)	0.481		
	AUC_0–5 h_	28.75 ± 22.7	45.10 ± 31.78	0.237		
VAS good effects (mm)	E_max_	32.56 ± 15.77	46.50 ± 30.05	0.242	**a.b**.**c**	**a.b**.**c**
	T_max_	0.33 (0.33–1.00)	0.33 (0.33–0.66)	0.743		
	AUC_0–5 h_	31.00 ± 23.19	43.85 ± 31.80	0.352		
VAS bad effects (mm)	E_max_	5.44 ± 7.23	16.25 ± 23	0.200	NS	NS
	T_max_	0.66 (0.00–2.00)	0.66 (0.33–2.00)	1.000		
	AUC_0–5 h_	6.03 ± 9.68	15.95 ± 27.90	0.331		
VAS liking (mm)	E_max_	37.67 ± 17.80	43.75 ± 33.23	0.639	**a.b**.**c**	**a.b.c**
	T_max_	0.33 (0.33–1.50)	0.33 (0.33–1.00)	0.963		
	AUC_0–5 h_	32.96 ± 26.41	43.91 ± 36.28	0.484		
VAS clarity (mm)	E_max_	35.11 ± 26.38	43.50 ± 36.34	0.591	**a.b**.**c**	**a.b.c**
	T_max_	0.66 (0.33–1.50)	0.50 (0.33–0.66)	0.606		
	AUC_0–5 h_	41.30 ± 41.87	52.77 ± 58.02	0.644		
VAS focused (mm)	E_max_	36.00 ± 25.48	45.13 ± 37.20	0.560	**a.b**.**c**	**a.b.c**
	T_max_	0.33 (0.33–1.50)	0.33 (0.33–0.66)	0.541		
	AUC_0–5 h_	43.93 ± 43.18	53.07 ± 55.03	0.707		
VAS open to others (mm)	E_max_	41.44 ± 21.74	41.88 ± 32.97	0.975	**a.b**.d	**a.b**
	T_max_	0.33 (0.33–1.50)	0.33 (0.33–0.66)	0.743		
	AUC_0–5 h_	39.30 ± 32.42	36.04 ± 35.95	0.847		
VAS trust to others (mm)	E_max_	33.78 ± 23.46	32.25 ± 34.68	0.916	**a.b**.d	**a.b**
	T_max_	0.33 (0.33–0.66)	0.66 (0.33–1.00)	0.139		
	AUC_0–4 h_	34.34 ± 34.92	28.20 ± 33.30	0.717		
VAS feeling close to others (mm)	E_max_	26.78 ± 18.61	30.88 ± 32.39	0.750	**a.b**.c	**a.b**
	T_max_	0.33 (0.33–1.00)	0.33 (0.33–0.66)	1.000		
	AUC_0–5 h_	29.96 ± 29.69	26.56 ± 31.59	0.822		
VAS would like to be with other people (mm)	E_max_	30.67 ± 24.62	36.50 ± 37.14	0.705	**a.b**.c	**a**.b
	T_max_	0.33 (0.00–0.66)	0.33 (0.00–2.00)	0.606		
	AUC_0–5 h_	31.98 ± 32.39	42.17 ± 58.68	0.659		
VAS would like to hug someone (mm)	E_max_	17.78 ± 23.69	18.63 ± 32.62	0.952	**a.b**	NS
	T_max_	0.33 (0.00–0.66)	0.66 (0.00–1.50)	0.321		
	AUC_0–5 h_	16.90 ± 23.44	20.15 ± 36.77	0.829		
VAS palpitations (mm)	E_max_	25.67 ± 17.96	27.13 ± 29.12	0.901	**a.b**	a.b
	T_max_	0.33 (0.00–1.00)	0.66 (0.00–1.50)	0.139		
	AUC_0–5 h_	25.44 ± 28.04	26.84 ± 39.61	0.934		
VAS anxiety (mm)	E_max_	10.22 ± 10.22	25.88 ± 31.53	0.178	b	NS
	T_max_	0.33 (0.00–1.50)	0.83 (0.33–2.00)	0.200		
	AUC_0–5 h_	9.55 ± 10.22	23.88 ± 41.56	0.331		
VAS sexual desire (mm)	E_max_	7.11 ± 16.09	6.50 ± 16.83	0.940	NS	NS
	T_max_	0.00 (0.00–1.00)	0.00 (0.00–1.00)	0.815		
	AUC_0–5 h_	18.44 ± 49.83	7.38 ± 19.29	0.565		
VAS sexual arousal (mm)	E_max_	1.56 ± 3.68	3.88 ± 10.18	0.532	NS	NS
	T_max_	0.00 (0.00–1.00)	0.00 (0.00–1.00)	1.000		
	AUC_0–5 h_	2.67 ± 6.95	4.63 ± 12.29	0.687		
ARCI PCAG (score)	E_max_	1.44 ± 4.45	−0.75 ± 4.46	0.327	NS	NS
	T_max_	2.00 (1.00–2.00)	1.00 (0.00–2.00)	0.236		
	AUC_0–5 h_	2.72 ± 10.46	−0.63 ± 6.14	0.442		
ARCI MBG (mm)	E_max_	7.00 ± 3.67	6.00 ± 4.47	0.620	**b.c.d**	**b**.**c**
	T_max_	0.66 (0.66–1.50)	0.66 (0.66–1.50)	1.000		
	AUC_0–5 h_	12.49 ± 10.75	6.57 ± 4.58	0.170		
ARCI LSD (score)	E_max_	1.67 ± 1.73	1.13 ± 3.04	0.654	c	NS
	T_max_	1.00 (1.0–2.0)	1.00 (1.00–3.00)	0.888		
	AUC_0–5 h_	0.56 ± 2.42	0.38 ± 5.13	0.926		
ARCI BG (score)	E_max_	3.56 ± 1.88	2.75 ± 2.87	0.499	c	**c**
	T_max_	1.00 (1.00–4.00)	1.00 (1.00–2.00)	0.743		
	AUC_0–5 h_	6.67 ± 7.13	4.75 ± 3.36	0.499		
ARCI A (score)	E_max_	3.78 ± 1.92	3.50 ± 2.07	0.778	**c.e**	**c.e**
	T_max_	1.00 (1.00–5.00)	1.00 (1.00–2.00)	0.673		
	AUC_0–5 h_	8.78 ± 6.25	6.31 ± 4.11	0.359		
VESSPA S (score)	E_max_	0.50 ± 0.62	0.40 ± 0.86	0.775	NS	NS
	T_max_	2.00 (0.00–5.00)	0.50 (0.00–2.00)	0.200		
	AUC_0–5 h_	1.22 ± 1.74	0.61 ± 1.25	0.424		
VESSPA ANX (score)	E_max_	1.15 ± 0.72	1.10 ± 0.79	0.904	**c.e**	**c.e**
	T_max_	1.00 (1.00–1.00)	1.00 (1.00–2.00)	0.200		
	AUC_0–5 h_	2.08 ± 1.51	1.79 ± 1.56	0.705		
VESSPA CP (score)	E_max_	0.06 ± 0.12	0.00 ± 0.00	0.202	NS	-
	T_max_	0.00 (0.00–1.00)	0.00	0.481		
	AUC_0–5 h_	0.07 ± 0.17	0.00 ± 0.00	0.234		
VESSPA SOC (score)	Emax	0.98 ± 1.08	0.79 ± 1.02	0.717	NS	**c**
	T_max_	1.00 (0.00–4.00)	1.00 (0.00–1.00)	0.481		
	AUC_0–5 h_	1.73 ± 2.23	0.98 ± 1.21	0.410		
VESSPA ACT (score)	E_max_	1.56 ± 1.12	1.42 ± 0.91	0.784	**c**	**c**
	T_max_	1.00 (1.00–4.00)	1.00 (0.00–1.00)	0.481		
	AUC_0–5 h_	2.66 ± 2.21	1.83 ± 1.16	0.359		
VESSPA PS (score)	E_max_	0.54 ± 0.41	0.27 ± 0.32	0.164	**c**	**c**
	T_max_	1.00 (0.00–4.00)	1.00 (0.00–2.00)	0.963		
	AUC_0–5 h_	0.88 ± 0.78	0.40 ± 0.60	0.174		

E_max_, peak effects 0–5 h (differences from baseline) measured by mm (visual analog scale [VAS]), and score (Addiction Research Center Inventory [ARCI], Evaluation of Subjective Effects of Substances with Abuse Potential questionnaire [VESSPA-SEE]) and expressed as mean ± standard deviation. A post-hoc Dunnett’s test for multiple comparisons was used. Statistical differences are presented as “a” *p* < 0.05, “**a**” *p* < 0.01 (times 0–0.33 h), “b” *p* < 0.05, “**b**” *p* < 0.01 (times 0–0.66 h), “c” *p* < 0.05, “**c**” *p* < 0.01 (times 0–1 h), “d” *p* < 0.05, “**d**” *p* < 0.01 (times 0–1.5 h), “e” *p* < 0.05, “**e**” *p* < 0.01 (times 0–2 h) and “f” *p* < 0.05, “**f**” *p* < 0.01 (times 0–2.5 h), “g” *p* < 0.05 “**g**” *p* < 0.01 (times 0–3 h), “h” *p* < 0.05, “**h**” *p* < 0.01 (times 0–4 h) and “i” *p* < 0.05, “**i**” *p* < 0.01 (times 0–5 h). NS, not significant. VASs, in italics are measured 0, 0.33, 0.66, 1, 1.5, 2, 2.5, 3, 4, 5 h. The other VASs, ARCI_PCAG/LSD/BG/A and VESSPA, at 0, 1, 2, 3, 4, 5 h, and ARCI MBG, at 0, 0.66, 1, 1.5, 2, 2.5, 3, 4, 5 h. For T_max_ data are reported, as median and range.

**FIGURE 2:**
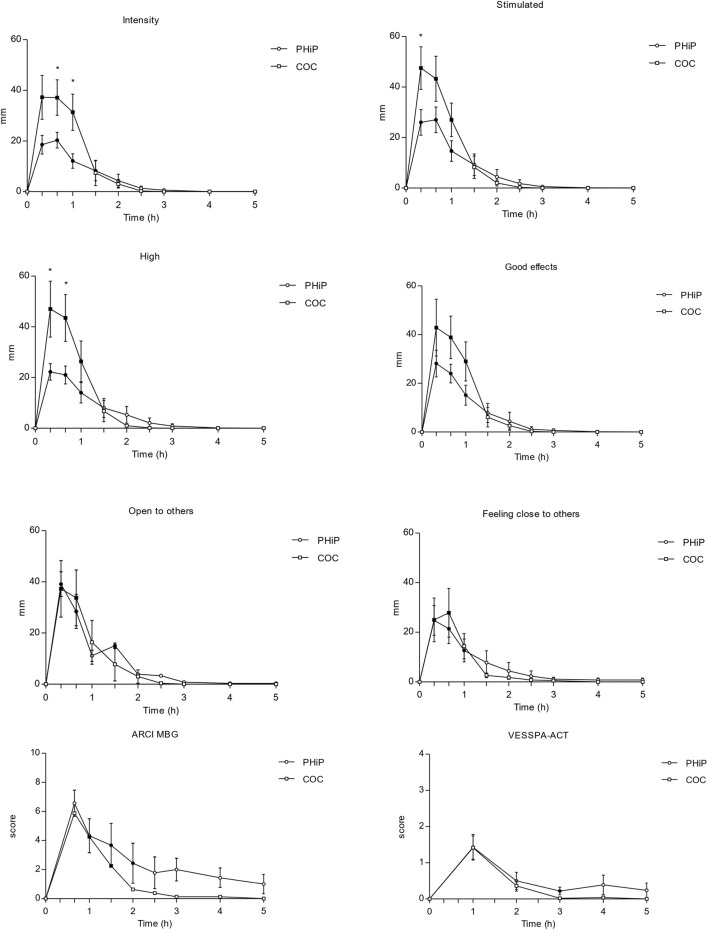
Time course (n = 17; mean ± standard error) of subjective effects following intranasal administration of α-PHiP (PHiP in the legend) and cocaine. Significant differences from the baseline are indicated with filled symbols • (p < 0.05). Includes intensity, stimulation, high, good effects, openness to others, feeling close to others, ARCI MBG and VESSPA-ACT. ARCI (Addiction Research Center Inventory questionnaire) subscale MBG (morphine-benzedrine group, euphoria) and VESSPA (Evaluation of Subjective Effects of Substances with Abuse Potential) subscale ACT (activity and energy).

In the ARCI questionnaire, significant differences from baseline were found in the MBG (morphine benzedrine group, euphoria), BG (benzedrine group, intellectual efficiency and energy), and A (amphetamine-like effects) subscales, in both substances. Figure 2 shows a selection of the main subjective effects reported with both substances.

Regarding the VESSPA-SSE, α-PHiP caused significant changes compared to baseline in several subscales, such as ANX (anxiety), SOC (pleasure and sociability), ACT (activity and energy), in similar proportion than with cocaine.

No serious adverse effects were reported. No changes were observed in the PANNS score during the sessions. Four participants reported an unpleasant sensation in the throat/numbness after snorting cocaine, which can be explained by its local anaesthetic effect.

### 3.3 α-PHiP, cocaine and cortisol in oral fluid

**FIGURE 3:**
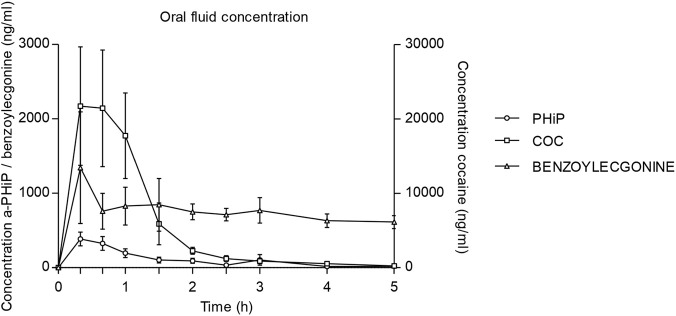
Time course of α-PHiP (PHiP in the legend), cocaine and its metabolite benzoylecgonine concentrations in oral fluid (75-90mg cocaine n=8 and 15-25mg α-PHiP n=9; mean, standard error).

Across all participants, α-PHiP was rapidly absorbed, and the time to reach maximum concentrations was shorter than for cocaine, 0.33 vs. 0.55 h, respectively (see Table 3). Mean concentration time curve for α-PHiP in OF is shown in Figure 3. After 5 h, α-PHiP concentrations were near-baseline levels. α-PHiP had median salivary half-life of 1.06 h (0.84–1.79, with an oulier of 5.83) while for cocaine it was 0.84 h and for BZE 3.22 h. Cocaine concentrations in OF were more than 5-fold higher than those of α-PHiP, while BZE concentrations were significantly lower in this matrix. Two volunteers had very high cocaine concentrations (>5,000 ng/mL) within the first hour suggesting contamination during the administration.

**TABLE 3:** Pharmacokinetic parameters of α-PHiP (*n* = 9) and cocaine (*n* = 8) in OF.

Oral fluid concentrations	α-PHiP	Cocaine
C_max_	451.25 ± 322.01	28753.61 ± 23183.86
T_max_	0.33 (0.33–0.66)	0.50 (0.33–1.00)
AUC_0-5 h_	534.39 ± 415.68	26375.75 ± 18357.40

Results of C_max_ (ng/mL) and AUC_0–5 h_ (ng/mL·h) are presented as mean ± standard deviation, T_max_ (h) is shown as median (min–max). AUC, area under the curve.

Although a slight increase in salivary cortisol was observed following administration, this change did not reach statistical significance across the study population, indicating that disposition in oral fluid occurred independently of measurable alterations in cortisol secretion dynamics.

### 3.4 α-PHiP urinary excretion

The total amount of α-PHiP excreted in urine over the 5-h period varied considerably among individuals, ranging from approximately 0.004–0.06 mg and only about 0.11% of the intranasally administered dose was excreted unchanged in urine. In detail, a total α-PHiP mean amount of 21.6 ± 16.8 µg was accumulated in urine up to 5 h, of which 6.4 ± 7.9 µg and 15.3 ± 16.1 µg between 0–2 and 2–5 h, respectively.

### 3.5 α-PHiP in sweat

Almost all volunteers exhibited very low or undetectable levels of α-PHiP in sweat samples collected over the 0–5 h interval. Only three participants demonstrated appreciable drug concentrations within this timeframe. One individual presented an exceptionally high value of 444.2 ng/patch, another exhibited a notably elevated level of 76.6 ng/patch, and a third showed a moderate concentration of 30.0 ng/patch. On average, approximately 0.09 ± 0.20 mg of α-PHiP was excreted in sweat after administration, equivalent to around 0.6% of the total intranasally administered dose.

### 3.6 α-PHiP in dry blood samples

α-PHiP levels in DBS samples collected 1 h after intranasal administration varied notably across volunteers. Most volunteers exhibited concentrations ranging from 8.3 to 24.1 ng/mL. However, one volunteer showed a markedly elevated concentration of 446.9 ng/mL, substantially exceeding values observed in all other participants.

## 4 Discussion

To the best of our knowledge, this is the first observational study under naturalistic conditions assessing the acute pharmacological effects of intranasal α-PHiP in humans, providing unique insights into its acute subjective and physiological effects, including comparisons with a control group of individuals using cocaine. Our primary findings demonstrate that α-PHiP exhibits characteristic psychostimulant effects, including pronounced cardiovascular responses such as increased heart rate, systolic blood pressure, and diastolic blood pressure. In comparison with cocaine, similar effects on heart rate have been observed between both substances. However, systolic and diastolic blood pressure showed a more sustained increase with α-PHiP than with cocaine. Regarding body temperature, the mild increase was only different from baseline for cocaine.

Regarding subjective effects, α-PHiP behaved similarly to cocaine, although the latter produced greater intensity and a stronger high, which could be related to the fact that users had more extensive experience with this substance. α-PHiP produced notable subjective effects, including feelings of stimulation, euphoria, openness to others, closeness to others, and enjoyment of the effect. These results are consistent with an empathogenic and psychostimulant profile and are comparable to those reported for other substances such as α-PVP, MDMA, mephedrone, and methylone, which are primarily empathogenic, as well as with substances with stronger psychostimulant effects, such as cocaine and amphetamines (Papaseit et al., 2016; Poyatos et al., 2023; de la Rosa et al., 2025). Hallucinogenic effects were not observed, and no changes in shapes, distances, or lights were reported. The description of the spectrum of effects produced by this synthetic cathinone is of primary importance for the individuation of the possible cause of the intoxications, which may support the clinical toxicologists to conduct specific analysis.

A similar observational study was conducted with orally administered methylone, in which participants self-administered doses ranging from 100 to 300 mg. The results revealed increases in heart rate along with typical stimulant and empathogenic effects (Poyatos et al., 2021; Papaseit et al., 2021). That study also compared methylone with MDMA following oral self-administration (75–100 mg), finding that MDMA produced comparable but less intense physiological and subjective effects. Likewise, a clinical trial assessing the oral administration of 200 mg methylone, 100 mg MDMA, and placebo demonstrated that both substances induced similar physiological and subjective responses. However, methylone produced a faster onset and a shorter duration of subjective effects compared to MDMA (Poyatos et al., 2023).

In the case of mephedrone, ten experienced drug users self-administered doses of 100–200 mg orally or 50–100 mg intranasally. The findings showed increases in systolic and diastolic blood pressure, temperature, and heart rate for both routes, with no significant differences in vital signs except for maximal cutaneous temperature (Poyatos et al., 2021).

A recently published crossover, placebo-controlled trial investigated the effects of oral administration of 3- methylmethcathinone (3-MMC) at doses of 25, 50, and 100 mg. Participants in that study reported mild increases in dissociative and psychedelic effects, which were not observed after consuming α- PHiP. Additionally, sympathomimetic effects previously described in this class of substances were also observed, resembling those of MDMA and amphetamines (Ramaekers et al., 2024).

The physiological and subjective effects observed with α-PHiP were quite similar to those previously reported for α-PVP, mephedrone, methylone and 3-MMC, but with greater intensity (Papaseit et al., 2016; Poyatos et al., 2023; Ramaekers et al., 2024; de la Rosa et al., 2025). The onset of effects and oral concentrations following intranasal α-PHiP self-administration occurred earlier than with oral administration of methylone and mephedrone, and was similar to that observed with intranasal α-PVP.

For α-PHiP, the intensity and stimulant effects peaked at 0.33 h, similar to that observed with α-PVP **(**de la Rosa. et al., 2025**),** while for cocaine occurred later, at 0.66 h. In the case of orally taken mephedrone (200 mg) and methylone (200 mg), the peak effects were observed at 0.75 h (Papaseit et al., 2021; Poyatos et al., 2023).

It is worth noting that neither α-PHiP, α-PVP, mephedrone, methylone, cocaine, nor MDMA induced hallucinations, psychotic episodes, or any other serious adverse events during the study sessions. The most likely explanation is that such effects are typically reported at higher doses—associated with intoxication—than those administered in the previous studies, which were low to moderate.

A systematic review conducted by Poyatos et al. (2022a) evaluated the pharmacological properties, abuse liability, and pharmacokinetics of various cathinones. The findings highlighted increases in blood pressure and heart rate, as well as subjective euphoria characterized by elevated energy and motor activation. The compounds examined included methylone, mephedrone, cathinone, and diethylpropion. Mechanistic studies indicate that mephedrone and methylone primarily elicit empathogenic effects, while pyrrolidine derivatives such as MDPV predominantly exhibit psychostimulant activity (Baumann et al., 2017; Baumann et al., 2018).

Research on other psychostimulants, most notably cocaine, has similarly shown that elevated heart rate and blood pressure are the main physiological responses. Cocaine is also known to induce euphoria, increased alertness and vigilance, and a reduced need for sleep (Farré et al., 1997). In the case of methamphetamine, another potent psychostimulant, intranasal doses ranging from 5 to 30 mg, have been associated with sympathomimetic effects, including rises in blood pressure, heart rate, and body temperature (Rush et al., 2011). α-PHiP administration, did not significantly increase cutaneous temperature, as happened with α-PVP.

All participants showed detectable concentrations of α-PHiP in oral fluid for up to 5 h. The rapid absorption observed in our study aligns with previous findings on structurally related pyrrolidinophenones such as α-PHP and α-PVP (Di Trana et al., 2025; Nuñez-Montero et al., 2023), confirming the efficient mucosal uptake and swift onset of action typical of these substances (Dinis et al., 2024; Nadal-Gratacós et al., 2024). The relatively short mean half-life observed in OF (1.1 h), may indicate a rapid decline in systemic concentrations. However, due to the influence of local physiological factors, OF kinetics should be interpreted with caution. Notably, the presence of an outlier with prolonged elimination highlights interindividual variability and underscores the need for further pharmacokinetic studies across multiple biological matrices, suggesting that oral fluid may be a suitable biological matrix for identifying recent α-PHiP use.

Approximately 0.11% of α-PHiP was excreted unchanged in urine within the first 5 h following administration. The lower urinary concentrations of the parent compound observed in our study, compared to previously reported levels of other cathinones after similar intranasal doses (Di Trana et al., 2025; Nuñez-Montero et al., 2023), may reflect more extensive metabolic transformation and potentially lower systemic bioavailability. As demonstrated in previous studies (Kemenes et al., 2022; Dinis et al., 2024; Yeh et al., 2024), α-PHiP metabolites are typically present in urine at higher concentrations as the parent drug. This limited renal excretion of unchanged α-PHiP emphasizes the importance of targeting metabolites for reliable detection, particularly in forensic and clinical toxicology.

Consistent with its metabolic profile, almost all volunteers exhibited very low or undetectable levels of α-PHiP in sweat samples collected over the 0–5 h interval. The very low levels detected in sweat further support the notion of extensive metabolism and/or limited excretion of the parent compound through this route. These results, however, are in agreement with the limited literature on cathinone excretion in sweat, where concentrations tend to be low and variable (Nuñez-Montero et al., 2023; Di Giorgi et al., 2023). The few individuals with higher sweat concentrations may reflect variable sweating rates, skin permeability, or dosing differences. Sweat patches provide limited utility for immediate post-consumption detection but might be valuable for assessing cumulative or repeated α-PHiP use.

DBS analysis revealed detectable levels of the analyte in most participants, although substantial interindividual variability was observed. One subject exhibited a notably high concentration, which may be attributed to differences in metabolism or sample collection. Excluding this outlier, α-PHiP concentrations in DBS were generally lower than in oral fluid, with OF/DBS ratios exceeding 1.4, consistent with previous findings reported for other cathinones or structurally related compounds (Gjerde et al., 2010; Sprega et al., 2023). The blood concentrations observed in this study (range 8.3–24.1 ng/mL) fall within the range previously reported in intoxication cases involving α-PiHP. For instance, Wachholz et al. (2023) described a fatal case in a 37-year-old male with α-PiHP and OH-α-PiHP concentrations ranging from 9 to 7,352 ng/mL (ng/g) and 18–310 ng/mL (ng/g), respectively, across different biological matrices. Similarly, another study reported α-PHiP concentrations between 7.33 and 118 μg/L in eight intoxicated patients (Brueckner I et al., 2024). These findings indicate that the concentrations measured in the present study are consistent with those documented in real-world intoxication scenarios. Importantly, the inclusion of DBS sampling represents a major strength of this study, as it demonstrates the feasibility of this minimally invasive, easily transportable, and storage-stable approach for the detection and quantification of synthetic cathinones (Wang et al., 2024; Morini et al., 2024).

The limitations of this study should be acknowledged. As an observational study, it is subject to inherent constraints, including potential selection bias favoring participants with extensive substance use experience, which may limit the generalizability of the findings to occasional or light users.

The small sample size and limited gender representation further restrict the ability to identify dose- or gender-specific effects. Although men reported stronger effects than women at higher doses, we were unable to directly compare responses between men and women receiving the same dose. These factors may also have influenced the statistical results, particularly in the application of Dunnett’s *post hoc* test, which may have lacked sufficient power to detect significant differences from baseline.

On the other hand, intense monitoring during the study could have resulted in stress-related effects. However, at the end of each session, participants were asked to describe their sensations in their own words, and none characterized the experience as stressful. This was further supported by the absence of an increase in cortisol concentrations. At least a third of them had participated in the past in other observational studies with other NPS. This fact reflects that in general they are willing to repeat. They explained a pleasant feeling after substances administration and pointed out that effects were similar to their previous uses of cocaine and amphetamines.

Regarding the observational design of this study and its potential limitations, it is important to note that the gold standard to evaluate subjective effects of substances is a randomized double-blind, placebo-controlled study. However**,** previous drug use/drug experience of participants can affect the validity of the blinding group due to the expectations of the effects based on previous consumption experience. In fact, when we evaluated participants with previous experience in psychostimulant consumption in two published double-blind and placebo-controlled clinical trials with MDMA, mephedrone and methylone (Papaseit et al., 2016; Poyatos et al., 2023), 92% and 94% of them, respectively, correctly recognized when they had received a placebo. Furthermore, between 83% and 94% of participants identified correctly the administered substance.

The subjective and physiological effects observed in other naturalistic/observational studies with psychostimulants and some psychedelics were very similar to those observed in double blind placebo-controlled studies. This overlap in the profile of pharmacological effects has been documented for MDMA (Papaseit et al., 2016; Khan et al., 2013; Poyatos et al., 2023; Angerer et al., 2024; Irvine et al., 2006; Morefield et al., 2011), mephedrone (Papaseit et al., 2016; Papaseit et al., 2021; Papaseit et al., 2020b; Freeman et al., 2012), methylone (Poyatos et al., 2023; Poyatos et al., 2021; Poyatos et al., 2022b), 4-bromo-2,5-dimethoxyphenethylamine (2C-B, Nexus) (Mallaroni et al., 2023; Papaseit et al., 2018), and 5-methoxy-N,Ndimethyltryptamine (5-MeO-DMT, mebufotenin) (Timmermann et al., 2025; Uthaug et al., 2019). The main effects were comparable in both methodological approaches, and only some variations in the intensity were detected. These findings reinforce the validity of observational studies conducted under standardized conditions, such as those presented here.

Another limitation of the present study was the lack of commercially available reference standards for α-PHiP metabolites, which prevented definitive identification and quantification of metabolism. The predominance of metabolites over the parent compound may have significant implications for the pharmacokinetic and pharmacodynamic properties of α-PHiP, potentially influencing its duration of action, potency, and toxicological profile (Paul et al., 2015; Grapp et al., 2023; Wachholz et al., 2023). Additionally, haematocrit was not determined in DBS samples**,** and its inclusion would likely have improved the robustness and accuracy of the drug concentration measurements.

Furthermore, although participants were instructed to abstain from consuming drugs of abuse during the week prior to the study and showed no clinical signs of intoxication at the start of the sessions, we cannot fully rule out prior use of α-PHiP. Baseline oral fluid samples were negative, which excludes very recent intake, but baseline urine screening rapid test performed was not able detect α-PHiP to discard an earlier consumption.

## 5 Conclusion

To the best of our knowledge, the present study is the first naturalistic study to assess the pharmacological effects of α-PHiP in humans. Intranasal administration of α-PHiP produces acute subjective and physiological effects that closely resemble those of the prototypical psychostimulant cocaine, highlighting its potential for abuse. Relative to cocaine, α-PHiP induced comparable subjective effects although intensity, high and stimulation feelings were lower while its impact on blood pressure was more prolonged. The results can be relevant for clinical toxicologists and doctors attending acute poisoning in emergency rooms. Future studies should include larger participant cohorts to confirm these findings and focus on full metabolic profiling.

## Data Availability

The raw data supporting the conclusion of this article will be made available by the authors, without undue reservation.
