# Supplementary material for: Disposition and effects of alpha-pyrrolidinoisohexanophenone (α-PHiP) in comparison with cocaine: an observational study
Source: Front Pharmacol. 2026 Jan 14;16:1728824. doi: 10.3389/fphar.2025.1728824 (PMC12848320; doi:10.3389/fphar.2025.1728824)
Supplement: Supplementary file 1 [file Supplementaryfile1.docx]

Supplementary Material

1. **Supplementary Tables**

- Supplementary Table 1 shows the validation parameters of α-PHiP
- Supplementary Table 2 shows the validation parameters of Cocaine (COC) and Benzoylecgonine (BZE)
- Supplementary Table 3 shows gender differences.

**Table 1** Linearity range, determination coefficient (R^2^), mandel’s fitting test (Fcri 95%), lower limit of detection (LOD), accuracy, precision, and recovery values for α-PiHP in OF, urine, sweat and DBS

| Matrix | Linearity range  (ng/mL or ng/patch) | LOD  (ng/mL or ng/patch) | R^2^ | Fcrit 95% | Accurancy  (BIAS%) | | | Intra-day precision  (CV%) | | | Inter-day precision  (CV%) | | | Recovery  (%) | | |
| --- | --- | --- | --- | --- | --- | --- | --- | --- | --- | --- | --- | --- | --- | --- | --- | --- |
|  |  |  |  |  | L | M | H | L | M | H | L | M | H | L | M | H |
| OF | 5-1000 | 1.5 | 0.994 | 3.3 | 8.2 | 15.5 | 10.2 | 2.6 | 10.0 | 5.3 | 5.7 | 5.5 | 2.7 | 100.6 | 102.1 | 100.4 |
| Urine | 5-500 | 1.5 | 0.997 | 2.1 | 13.6 | 13.8 | 13.5 | 7.7 | 12.3 | 12.3 | 10.6 | 13.5 | 12.1 | 87.4 | 91.1 | 89.5 |
| Sweat | 5-500 | 1.5 | 0.990 | 1.6 | 14.0 | 11.2 | 7.6 | 17.5 | 10.3 | 5.6 | 11.8 | 7.7 | 3.3 | 93.7 | 90.0 | 101.9 |
| DBS | 5-500 | 1.5 | 0.959 | 1.9 | 8.8 | 10.6 | 14.2 | 7.6 | 14.2 | 17.0 | 3.9 | 11.2 | 16.5 | 102.9 | 101.9 | 94.5 |

Abbreviation: OF = Oral Fluid; DBS = Dried Blood Spot, L = low quality control concentration (7.5 ng/mL for OF and urine, 7.5 ng/patch for sweat and 7.5 ng/DBS for dried blood spot); M = medium control concentration (250 ng/mL for OF and urine, 250 ng/patch for sweat and 250 ng/DBS for dried blood spot); H = high control concentration (850 ng/mL for OF and urine, 450 ng/patch for sweat and 450 ng/DBS for dried blood spot)

**Table 2** linearity range, determination coefficient (R2), mandel’s fitting test (Fcri 95%), lower limit of detection (LOD), accuracy, precision, and recovery values for Cocaine (COC) and Benzoylecgonine (BZE) in OF, urine and sweat

| Matrix | Linearity range  (ng/mL or ng/patch) | LOD  (ng/mL or ng/patch) | R^2^ | Fcrit 95% | Accurancy  (BIAS%) | | | Intra-day precision  (CV%) | | | Inter-day precision  (CV%) | | | Recovery  (%) | | |
| --- | --- | --- | --- | --- | --- | --- | --- | --- | --- | --- | --- | --- | --- | --- | --- | --- |
| COC | | | | | | | | | | | | | | | | |
|  |  |  |  |  | L | M | H | L | M | H | L | M | H | L | M | H |
| OF | 10-1000 | 1.5 | 0.994 | 3.1 | 7.1 | 10.3 | 8.89 | 7.8 | 6.9 | 6.2 | 9.5 | 8.7 | 8.0 | 94 | 97 | 101 |
| Urine | 10-1000 | 1.5 | 0.997 | 4.9 | 6.2 | 4.7 | 9.8 | 8.3 | 7.1 | 6.8 | 10.3 | 9.6 | 8.9 | 92 | 95 | 99 |
| Sweat | 10-500 | 1.5 | 0.990 | 4.2 | 10.1 | 5.3 | 5.1 | 9.7 | 9.5 | 5.4 | 10.3 | 10.2 | 10.1 | 90 | 93 | 98 |
| BZE | | | | | | | | | | | | | | | | |
|  |  |  |  |  | L | M | H | L | M | H | L | M | H | L | M | H |
| OF | 1-1000 | 0.3 | 0.993 | 4.2 | 3.4 | 2.8 | 5.1 | 6.5 | 9.5 | 5.4 | 8.2 | 7.5 | 7.0 | 96 | 98 | 102 |
| Urine | 1-1000 | 0.3 | 0.995 | 3.5 | 4.2 | 3.7 | 6.3 | 7.1 | 6.4 | 6.0 | 9.0 | 8.1 | 7.6 | 95 | 97 | 101 |
| Sweat | 1-500 | 0.3 | 0.990 | 4.7 | 5.0 | 4.1 | 7.2 | 8.9 | 10.0 | 7.3 | 10.0 | 10.2 | 9.5 | 93 | 96 | 100 |

Abbreviation: OF = Oral Fluid; L = low quality control concentration (15 ng/mL or 15 ng/patch for COC and 2.5 ng/mL or ng/patch for BZE); M = medium control concentration (250 ng/mL or 150 ng/patch for COC and 250 ng/mL or 85 ng/patch for BZE); H = high control concentration (850 ng/mL or 450 ng/patch for COC and BZE)

**Table 3**. Gender differences of the study participants

|  |  | Mean ± SD | | ANOVA |
| --- | --- | --- | --- | --- |
|  |  | **Men** | **Woman** | ***p* value** |
| Subjective effects |  |  |  |  |
| Temperature | Emax | 37.08±0.39 | 36.92±0.19 | 0.001 |
|  | AUC0-5 h | 183.98±1.28 | 183.86±1.00 | 0.001 |
| VAS intensity | AUC0-5 h | 26.07±24.00 | 24.83±12.97 | 0.041 |
| VAS high | AUC0-5 h | 32.34±31.88 | 25.88±15.66 | 0.012 |
| VAS good effects | AUC0-5 h | 34.90±34.38 | 27.87±12.70 | 0.005 |
| VAS liking | AUC0-5 h | 41.22±38.09 | 26.34±13.57 | 0.003 |
| VAS clarity | AUC0-5 h | 44.42±56.01 | 38.80±33.69 | 0.029 |
| VAS focused | AUC0-5 h | 47.13±56.71 | 41.38±36.03 | 0.033 |
| Changes in colors | Emax | 0.75±1.50 | 0.00±0.00 | <0.001 |
|  | AUC0-5 h | 0.75±1.50 | 0.00±0.00 | <0.001 |
| Changes in shapes | Emax | 0.75±1.50 | 0.00±0.00 | <0.001 |
|  | AUC0-5 h | 0.75±1.50 | 0.00±0.00 | <0.001 |
| VAS unreal body feeling | Emax | 2.00±4.00 | 0.00±0.00 | <0.001 |
|  | AUC0-5 h | 4.00±8.00 | 0.00±0.00 | <0.001 |
| VAS open to others | AUC0-5 h | 43.31±44.47 | 36.09±24.30 | 0.020 |
| VAS feeling close to others | AUC0-5 h | 36.27±41.13 | 24.12±19.97 | 0.011 |
| VAS be with other people | AUC0-5 h | 37.73±41.47 | 27.38±27.37 | 0.039 |
| VAS palpitations | AUC0-5 h | 31.94±41.15 | 20.24±15.04 | 0.002 |
| VAS sexual desire | Emax | 14.75±23.31 | 1.00±2.24 | <0.001 |
|  | AUC0-5 h | 40.25±73.98 | 1.00±2.24 | <0.001 |
| VAS sexual arousal | Emax | 3.53±5.20 | 0.00±0.00 | <0.001 |
|  | AUC0-5 h | 6.00±10.10 | 0.00±0.00 | <0.001 |
| ARCI-MBG | Emax | 9.00±4.69 | 5.40±1.82 | 0.009 |
|  | AUC0-5 h | 16.31±15.57 | 9.43±4.80 | 0.003 |
| ARCI-BG | Emax | 8.25±2.50 | 7.00±1.22 | 0.004 |
|  | AUC0-5 h | 30.00±9.70 | 24.00±3.34 | 0.001 |
| ARCI-A | Emax | 3.75±2.87 | 3.80±1.10 | 0.007 |
|  | AUC0-5 h | 30.00±9.70 | 24.00±3.34 | 0.002 |
| VESSPA-ANX | AUC0-5 h | 2.75±1.88 | 1.54±1.06 | 0.044 |
| VESSPA-ACT | AUC0-5 h | 2.86±2.98 | 2.50±1.74 | 0.021 |

Only those outcomes showing gender differences in the two-way ANOVA are included in the table
